# Supplementary material for: Competition between Hydration Shell and Ordered Water Chain Induces Thickness-Dependent Desalination Performance in Carbon Nanotube Membrane
Source: Membranes (Basel). 2023 May 18;13(5):525. doi: 10.3390/membranes13050525 (PMC10220983; doi:10.3390/membranes13050525)
Supplement: Supplementary file 1 [file membranes-13-00525-s001.zip › membranes-2383603-supplementary.pdf]

membranes

MDPI

Supplementary Materials

Competition Between Hydration Shell and Ordered Water Chain Induces Thickness-Dependent Desalination Performance in Carbon Nanotube Membrane

Siyi Liu [1], Liya Wang [1,*], Jun Xia [1], Ruijie Wang [1], Chun Tang [1] and Chengyuan Wang [2,*]

1 Faculty of Civil Engineering and Mechanics, Jiangsu University, Zhenjiang 212013, China
2 Zienkiewicz Centre for Computational Engineering, Faculty of Science and Engineering, Swansea University, Bay Campus, Swansea SA1 8EN, UK

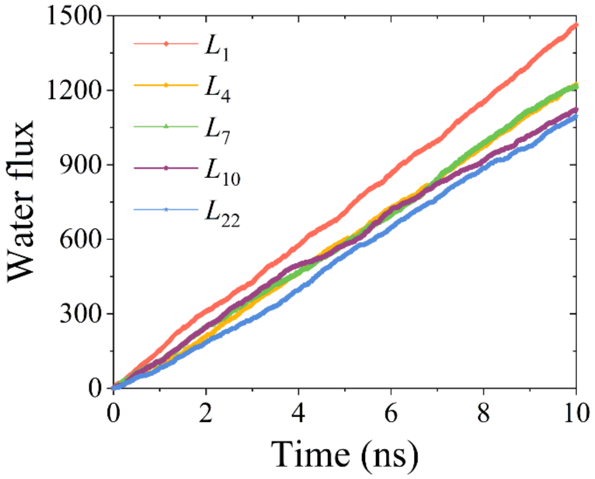

Figure S1. Water flux of thickness-variant (7, 7) CNTs versus simulation time.

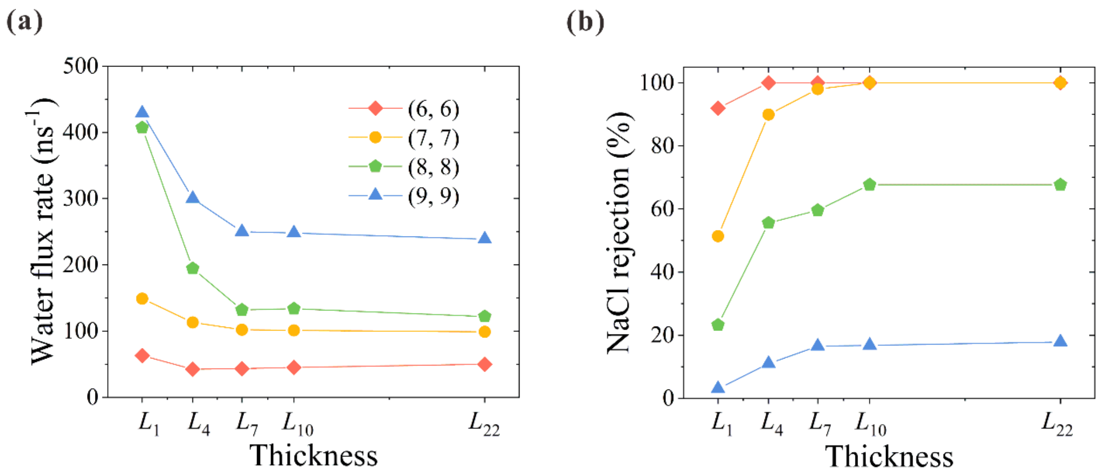

Figure S2. Thickness-dependent (**a**) water flux rate and (**b**) NaCl rejection in (6, 6), (7, 7), (8, 8) and (9, 9) CNTs.
